# Efficacy and side effect of transcatheter arterial embolization using a novel embolic agent for lateral epicondylitis in a diabetic patient: A case report

**DOI:** 10.1097/MD.0000000000047390

**Published:** 2026-01-30

**Authors:** Ha-Min Kim, Kun Yung Kim, Myoung-Hwan Ko, Gi-Wook Kim

**Affiliations:** a Jeonbuk National University Medical School, Jeonju, Republic of Korea; b Department of Radiology, Seoul National University Bundang Hospital, Seongnam, Republic of Korea; c Department of Physical Medicine & Rehabilitation, Jeonbuk National University Medical School, Jeonju, Republic of Korea; d Research Institute of Clinical Medicine of Jeonbuk National University – Biomedical Research Institute of Jeonbuk National University Hospital, Jeonju, Republic of Korea.

**Keywords:** absorbable gelatin sponge, diabetes mellitus, lateral epicondylitis, pain management, therapeutic embolization

## Abstract

**Rationale:**

Lateral epicondylitis (LE) is an overuse syndrome of the extensor carpi radialis brevis tendons of the forearm, leading to pain around the lateral epicondyle of the humerus exacerbated by several activities and a decrease in work productivity. This case study reports the efficacy and side effects of transcatheter arterial embolization (TAE) using Smartgel MIRIP, a novel quick-soluble gelatin sponge particles agent, through a LE patient with diabetes mellitus (DM).

**Patient concerns:**

The 61-year-old male presented with right elbow pain persisting for a month, which was exacerbated during wrist extension and made him unable to actively lift the wrist.

**Diagnoses and interventions:**

Ultrasound and magnetic resonance imaging confirmed LE with partial tearing of the common extensor tendon and associated cystic lesions. Given the patient’s DM, adverse effects from steroid injections, and poor response to conservative therapies, TAE with Smartgel MIRIP was performed as an alternative intervention.

**Outcomes:**

The visual analogue scale, patient-rated tennis elbow evaluation, and quick disabilities of the arm, shoulder and hand questionnaires were administered to evaluate patient’s pain and function. Transient post-procedural pain aggravation, dysfunction, diffuse ecchymosis, and swelling were observed but improved within days. Pain relief and functional improvement compared to baseline were observed from the 1 week after TAE and following periods as measured by visual analogue scale, patient-rated tennis elbow evaluation, and quick disabilities of the arm, shoulder, and hand.

**Lessons:**

This study focused on the embolic agent of TAE suggesting Smartgel MIRIP as an alternative to imipenem-cilastatin sodium without antimicrobial restriction. In addition, our report proposed DM as an important clinical history factor that can impact outcomes in TAE.

## 1. Introduction

Lateral epicondylitis (LE) is an overuse syndrome of the extensor carpi radialis brevis tendons of the forearm. In clinical care, it is one of the most frequent overuse syndromes. LE, associated with repetitive and forceful activity, is evoked by excessive stress, repeated microtrauma, and degenerative changes. A major complaint of patients with LE is the pain around the lateral epicondyle of the humerus, which is exacerbated by several activities. Some patients even experience disrupted work productivity by LE.^[1–3]^ Persisting pain is associated with increased disability, reduced quality of life, and a poorer prognosis.^[4,5]^

Primary treatment for LE is rest with nonsteroidal anti-inflammatory drugs, followed by physiotherapy if symptoms persist.^[6–8]^ Brace for decrease stress, extracorporeal shock wave therapy for analgesia, and prolotherapy for repair damaged fibers are also reported as one of LE treatments.^[3]^ Recently, injections of platelet-rich plasma and autologous blood were proposed as treatments for LE to promote tendon regeneration.^[9]^ Surgical treatment is considered when those conservative treatment is failed.^[8]^

Recently, several studies have reported the use of transcatheter arterial embolization (TAE) for musculoskeletal pain.^[10]^ The therapeutic mechanism of TAE involves inhibiting angiogenesis-driven sensory nerve growth, as these nerves frequently proliferate alongside newly formed blood vessels under the influence of proangiogenic factors such as vascular endothelial growth factor, beta-nerve growth factor, and various neuropeptides.^[11–13]^ TAE may also effectively alleviate pain associated with LE, as previous study demonstrated that neovessels and the accompanying nerves are causes of LE pain through atypical fibrous granulation containing numerous small blood vessels and perivascular innervation in biopsied tissue.^[12]^ While several studies have examined TAE for LE treatment primarily using imipenem-cilastatin sodium (IMP-CS) as the embolic agent, no previous research has evaluated quick-soluble gelatin sponge particles (QS-GSP), which have recently been proposed as an alternative to IMP-CS.^[14,15]^

This case reports the efficacy and temporal side effects of TAE in LE with Smartgel MIRIP, a novel QS-GSP agent, presenting a patient with diabetes mellitus (DM) who is refractory to conservative treatment and has limitations in steroid injections.

## 2. Case description

**Figure 1.**
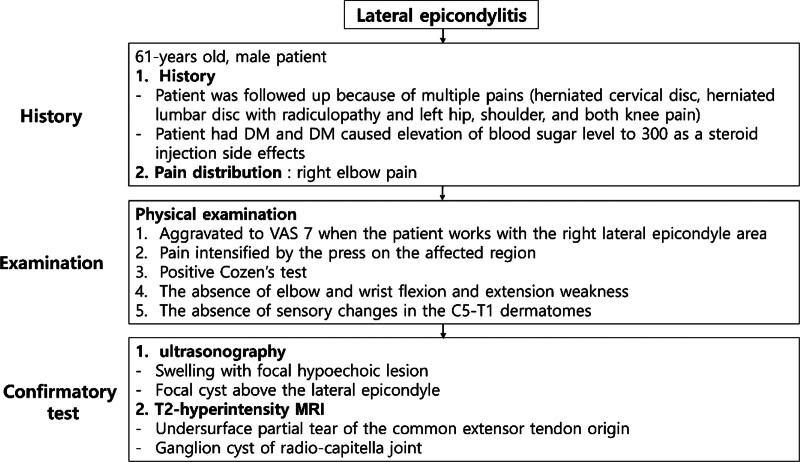
Flow chart of lateral epicondylitis. DM = diabetes mellitus, VAS = visual analogue scale.

A 61-year-old male patient presented with right elbow pain for a month (Fig. 1). The patient had a lot of physical activity due to orchard management. He was followed up by the department of rehabilitation in our hospital for medication and physical therapy of multiple pains (herniated cervical disc, herniated lumbar disc with radiculopathy and left hip, shoulder, and both knee pain). He had a history of DM and DM caused elevation of blood sugar level to 300 as a steroid injection side effect.

**Figure 2.**
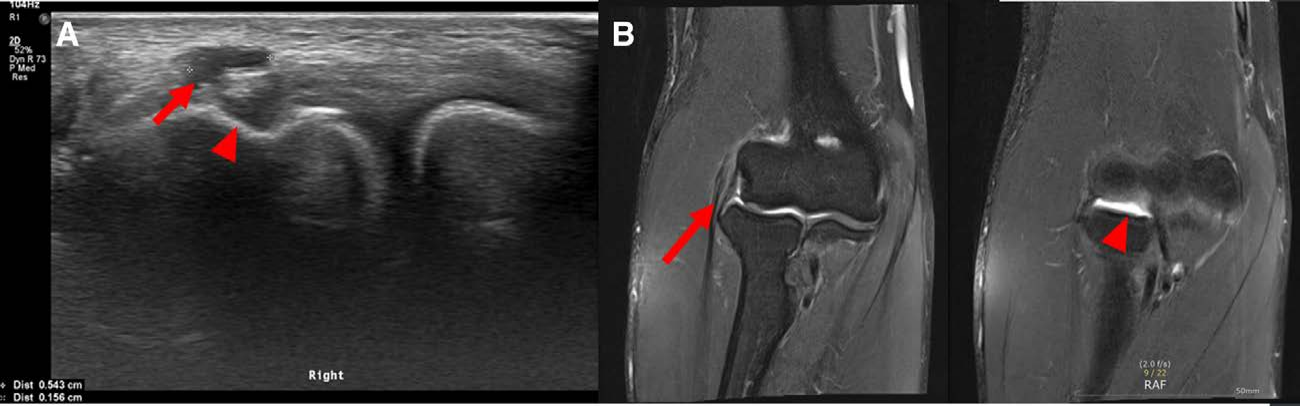
US shows swelling with focal hypoechoic lesion (red arrow A) and focal cyst above inferior lateral epicondyle (red arrowhead A) MRI shows T2-hyperintensity compatible with an undersurface partial tear of the common extensor tendon origin (red arrow B), and ganglion cyst of radio-capitellar joint (red arrowhead B). MRI = magnetic resonance imaging, US = ultrasound.

Right elbow pain newly emerged during multiple pains follow up. The symptoms made his daily life impossible and even aggravated to visual analogue scale (VAS) 7 when the patient works with the right lateral epicondyle area. The pain intensified by the press on the affected region. Right elbow pain was diagnosed as LE and ganglion cyst of radio-capitellar joint by ultrasound (US) (Fig. 2A) and magnetic resonance imaging (Fig. 2B). US shows swelling with focal hypoechoic lesion, and focal cyst above the lateral epicondyle. Magnetic resonance imaging shows T2-hyperintensity compatible with an undersurface partial tear of the common extensor tendon origin, and ganglion cyst of radio-capitellar joint. Cozen test was positive, and the patient exhibited severe pain during wrist extension, being unable to actively lift the wrist. Apart from this pain-induced limitation, no additional weakness was noted in elbow flexion/extension and or wrist flexion. Furthermore, there were no sensory changes in the C5 to T1 dermatomes, which supports exclusion of radiculopathy or peripheral nerve entrapment in the differential diagnosis.

**Figure 3.**
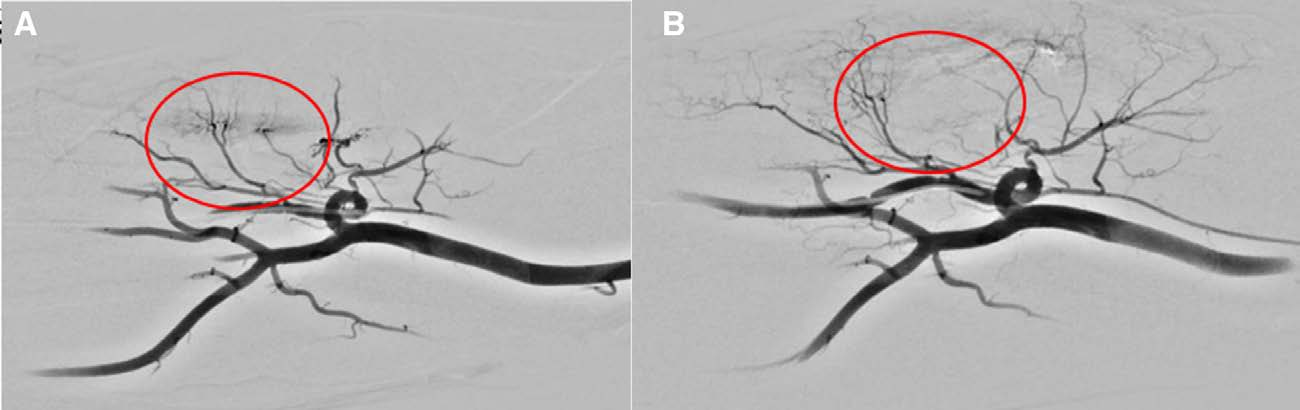
Angiography during TAE shows linear hypervascular staining from recurrent radial artery branch (red circle A). After embolization angiography shows complete devascularization of hypervascular staining (red circle B). TAE = transcatheter arterial embolization.

After consent from the patient, he was hospitalized, and we decided to perform TAE as treatment because of limitation in steroid injection and resistance to both medication therapy and physical therapy for LE. TAE was performed by an experienced interventional radiologist through an angiography. The US guided right radial access was performed with 5-Fr sheath (Prelude; Merit Medical, South Jordan). Angiography showed linear hypervascular staining from right recurrent radial artery branch, which corresponds to area of palpable tenderness (Fig. 3A). Right recurrent radial artery was superselected with 1.7Fr microcatheter (Veloute; Asahi Intecc, Aichi, Japan), then approximately 1 mL of quick soluble gelfoam (Smartgel MIRIP 50–125 µm, PL Micromed, Yangsan-si, Korea) mixed with iodine contrast media (Visipaque; GE Healthcare, Cork, Ireland) was injected until complete stasis was achieved. Completion angiography showed devascularization of the hypervascular staining (Fig. 3B).

The patient’s LE pain was measured by the VAS. Patient’s pain intensity from 0 “no pain at all” to 10 “worst pain imaginable,” presented in horizontal line, was measured by the patient marks.^[16,17]^ VAS was evaluated 1 day before, 1 day after, 1 week after, 3 weeks after, and 1 month after the TAE. Although the side effect of the worsening elbow pain VAS 7 to VAS 9 was reported the next day after the procedure temporarily, gradual alleviation of pain relative to VAS degree before TAE was observed thereafter. He showed VAS 5 at 1 week after TAE, VAS 3 at 3 weeks after TAE, and finally VAS 2 at 1 month after TAE (Table 1). Patient-rated tennis elbow evaluation (PRTEE) evaluated pain and function disability and quick disabilities of the arm, shoulder and hand (Quick-DASH) was used for functional evaluation. PRTEE consists of 5 items evaluating pain and 10 items evaluating disability, and each item is scored from 0 to 10, totally computed on a scale of 100 (0 = no disability, and 100 = sever disability). It is used as a disease-specific outcome measure.^[18]^ Quick-DASH, extracted from full-length DASH, is calculated by 11 items. Each item has 5 response option and Quick-DASH scored from 0 to 100, on which point 0 indicates no disability and point 100 indicates most severe disability.^[19]^ PRTEE and Quick-DASH were evaluated at the same time as VAS, showing similar course of change. PRTEE score was elevated from 41 to 76 at 1 day after TAE, but improved to 28 on 1 week after TAE, 17 on 3 weeks after TAE, and 9 on 1 month after the procedure. The patient showed transient increase in Quick-DASH score 31.8 to 56.8 on 1 day after, however recovered to 20.5 at 1 week after, 11.4 at 3 weeks after, and 4.5 at 1 month after the procedure (Table 1).

**Table 1:** Summary of outcome after TAE.

Outcome measures	Baseline	1 day after	1 week after	3 weeks after	1 month after
VAS	7	9	5	3	2
PRTEE					
Pain	26	32	17	9	4
Function disability	15	44	11	8	5
Total	41	76	28	17	9
Quick-DASH	31.8	56.8	20.5	11.4	4.5

PRTEE = patient-rated tennis elbow evaluation, TAE = transcatheter arterial embolization, VAS = visual analogue scale.

**Figure 4.**
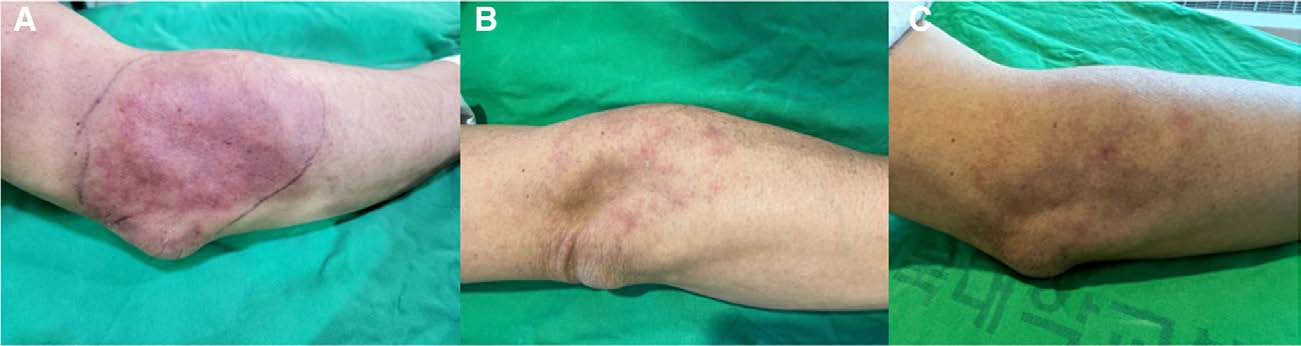
(A) Diffuse ecchymosis 1 day after TAE, (B) petechiae 6 day after TAE, (C) petechiae 1 week after TAE. TAE = transcatheter arterial embolization.

Skin side effects, diffuse ecchymosis, and swelling, were reported immediately after the TAE. Therefore, cold therapy and daily follow up were performed. As a result, swelling and ecchymosis showed daily improvement from the second day after the procedure (Fig. 4).

## 3. Discussion and conclusion

This case report aimed to show the efficacy and transient side effects of TAE with a new embolic agent from the LE patient who had DM. Although temporarily aggravated pain, decreased function, and skin symptoms such as diffuse ecchymosis and swelling were reported after procedure, the effectiveness of TAE had proven as VAS, PRTEE, and Quick-DASH showed pain relief and functional improvement thereafter.

LE is defined as degenerative process tendinosis which is evoked when the rate of stretching exceeds the tolerance of the tendon micro-tear results. A major complaint from LE patients is pain of lateral epicondyle area that often radiates extensor muscle and upper arm. Intensity of the pain is various, but severe pain can constrict daily activities and even disturb sleeping.^[20]^ The phase of LE is determined by description of level of pain.^[21]^ Treatment of LE are aimed to control pain, preserve movement, improve in grip strength and endurance, and finally return to normal function during controlling further histological and clinical deterioration.^[20]^ Rest with nonsteroidal anti-inflammatory drugs is considered as first-line treatment of LE and secondary treatment options for LE include physiotherapy, bracing, platelet–rich plasma injections, and corticosteroids injections, among others.^[6]^ Surgery is also considered when those nonoperative treatment are failed.^[22]^ Although many treatment options have been advocated, up to 50% of patients are refractory to conservative treatment and the optimal approach remains unclear.^[21,23]^ Meanwhile, TAE, traditionally used to control bleeding, is deployed for musculoskeletal pain, and more recently, its use for LE has emerged, showing good outcome.^[10,12,13]^

Several studies have reported the use of IMP-CS in TAE due to its temporary embolic effect, demonstrating safety and effectiveness in relieving pain associated with tendinopathies including LE.^[10,12,13,24]^ However, IMP-CS has practical limitations because it is primarily an antimicrobial agent, making it unavailable in many countries. Recently, QS-GSP has emerged as a promising alternative to IMP-CS, as it is specifically developed for temporary embolization purpose without antimicrobial restrictions and has demonstrated safety and effectiveness in several studies.^[13,14]^ Accordingly, Smartgel MIRIP (50–125 µm), a newly developed QS-GSP agent that dissolves spontaneously within several hours, was utilized in this case report with TAE treatment. However, the proper composition, dissolution time, and particle size of QS-GSP remain unclear. Further studies are required to establish the optimal form of QS-GSP and to clarify its degradation process, and potential complications. The economic implications of TAE should be interpreted within the context of each healthcare system. In terms of cost, a direct comparison between transarterial genicular artery embolization and other minimally invasive options, such as intra-articular injection therapies, is inherently limited by the marked variability in institutional pricing and reimbursement policies. Importantly, TAE is not covered by national insurance systems in most countries, and its procedural costs are largely borne by hospitals or patients. This contrasts with injection-based therapies, which are generally reimbursed and associated with lower initial expenses. While QS-GSP requires angiography suite resources, microcatheters, and embolic agents, resulting in higher upfront costs, injection therapies may necessitate multiple sessions over time, which may narrow the overall cost gap. Given these differences, systematic cost-effectiveness evaluations of TAE within specific national healthcare systems are warranted to clarify its economic value in real-world practice.

In our study, a 61-year-old LE patient who was suffering other multiple pains was treated with TAE by Smartgel MIRIP. TAE was performed due to his history of DM which caused sugar level to 300 following steroid injection and due to his resistance to both medication and physical therapy for LE. Outcomes of procedure were evaluated based on pain and functional parameters. VAS, PRTEE, and Quick-DASH were improved at 1 week after TAE with continued enhancement at 3 weeks and 1 month after TAE, despite worsening in 1 day after. As musculoskeletal pain is evoked by abnormally hypervascular or inflamed tissue, pain resolution is attained by blocking abnormal vascular supply. TAE makes pathologic tissue no longer be a nociceptive trigger or produce pro-inflammatory mediators.^[10]^

There were temporal adverse effects. Immediately after TAE, the patient reported worsening index in VAS, PRTEE, and Quick-DASH, as well as diffuse ecchymosis and swelling. Although previous studies reported minor adverse effects such as erythema after TAE, the adverse effects appeared somewhat more pronounced in the present study.^[10,23]^ These complications may have been induced by the degradation products of the spherical fish gelatin component of Smartgel MIRIP causing embolization of cutaneous branches. Although IMP-CS particles are smaller (approximately 10–70 µm) compared to Smartgel MIRIP, their actual dissolution may be faster under pulsatile flow conditions. Another possible explanation is the patient’s history of DM. DM could have contributed to the adverse effects by reducing peripheral circulation, potentially increasing susceptibility to ischemic complications. DM impairs arteriogenesis and influences the recruitment and dilatation of collateral arteries, growth factor signaling, and various remodeling processes.^[25]^ Accordingly, DM may significantly influence clinical outcomes following TAE. However, few studies have directly addressed this clinical challenge, and our case involving a LE patient with DM highlights its clinical importance.

This case report performed TAE by Smartgel MIRIP, novel QS-GSP agent, to LE patient with DM. Although temporal adverse effects such as skin symptoms, deteriorated pain, and decreased function were showed immediately after procedure, alleviated skin symptoms, pain relief, and functional improvement were reported from 1 week after TAE. The present study provided valuable insights into the effects of TAE in patients with DM and alternative possibilities in QS-GSPs with new agent Smartgel MIRIP. To confirm and expand this report, further continuous research is needed.

## Acknowledgments

The authors extend their appreciation to all members of the Department of Physical Medicine & Rehabilitation and Radiology at Jeonbuk National University Hospital.

## Author contributions

**Conceptualization:** Kun Yung Kim, Gi-Wook Kim.

**Data curation:** Kun Yung Kim, Gi-Wook Kim.

**Funding acquisition:** Myoung-Hwan Ko.

**Investigation:** Kun Yung Kim, Myoung-Hwan Ko, Gi-Wook Kim.

**Methodology:** Kun Yung Kim, Gi-Wook Kim.

**Project administration:** Gi-Wook Kim.

**Resources:** Kun Yung Kim, Gi-Wook Kim.

**Software:** Ha-Min Kim.

**Supervision:** Gi-Wook Kim.

**Validation:** Kun Yung Kim, Myoung-Hwan Ko.

**Visualization:** Ha-Min Kim, Kun Yung Kim, Gi-Wook Kim.

**Writing – original draft:** Ha-Min Kim.

**Writing – review & editing:** Ha-Min Kim, Kun Yung Kim, Gi-Wook Kim.
